# Influenza co-infection associated with severity and mortality in COVID-19 patients

**DOI:** 10.1186/s12985-021-01594-0

**Published:** 2021-06-14

**Authors:** Bandar Alosaimi, Asif Naeem, Maaweya E. Hamed, Haitham S. Alkadi, Thamer Alanazi, Sanaa Saad Al Rehily, Abdullah Z. Almutairi, Adnan Zafar

**Affiliations:** 1 Department of Research Labs, Research Center, King Fahad Medical City, P.O. Box. 59046, Riyadh, 11525 Saudi Arabia; 2 Department of Botany and Microbiology, College of Science, King Saud University, Riyadh, Saudi Arabia; 3 Department of Pathology and Laboratory Medicine, King Abdullah Bin Abdulaziz University Hospital, Princess Nourah University, Riyadh, Saudi Arabia; 4 Infection Diseases Department, King Fahad Hospital, Medina, Saudi Arabia; 5 Laboratory and Blood Bank Department, King Fahad Hospital, Medina, Saudi Arabia; 6 Pediatric Pulmonology Department, King Fahad Medical City, Riyadh, Saudi Arabia

**Keywords:** Co-infection, COVID-19, Influenza A H1N1, Mortality, SARS-CoV-2

## Abstract

**Background:**

In COVID-19 patients, undetected co-infections may have severe clinical implications associated with increased hospitalization, varied treatment approaches and mortality. Therefore, we investigated the implications of viral and bacterial co-infection in COVID-19 clinical outcomes.

**Methods:**

Nasopharyngeal samples were obtained from 48 COVID-19 patients (29% ICU and 71% non-ICU) and screened for the presence of 24 respiratory pathogens using six multiplex PCR panels.

**Results:**

We found evidence of co-infection in 34 COVID-19 patients (71%). Influenza A H1N1 (n = 17), *Chlamydia pneumoniae* (n = 13) and human adenovirus (n = 10) were the most commonly detected pathogens. Viral co-infection was associated with increased ICU admission (r = 0.1) and higher mortality (OR 1.78, CI = 0.38–8.28) compared to bacterial co-infections (OR 0.44, CI = 0.08–2.45). Two thirds of COVID-19 critically ill patients who died, had a co-infection; and Influenza A H1N1 was the only pathogen for which a direct relationship with mortality was seen (r = 0.2).

**Conclusions:**

Our study highlights the importance of screening for co-infecting viruses in COVID-19 patients, that could be the leading cause of disease severity and death. Given the high prevalence of Influenza co-infection in our study, increased coverage of flu vaccination is encouraged to mitigate the transmission of influenza virus during the on-going COVID-19 pandemic and reduce the risk of severe outcome and mortality.

## Background

The newly emerged Severe Acute Respiratory Syndrome Coronavirus 2 (SARS-CoV-2) continues to circulate outside of Wuhan, China since December 2019, and now exported to different countries all over the world [1]. At the time of writing this report, there were nearly a quarter of a million of Coronavirus Disease-19 (COVID-19) confirmed cases ranking Saudi Arabia as the 14th highest in the world [2]. Most hospitalized patients needed admission to intensive care unit (ICU) and mortality reaches up to 50% in some cases [3]. Until now, twenty-two studies have reported co-infection in COVID-19 and of these 16 have evidence of viral co-infection [4]. The prevalence of critical cases with viral co-infection has been reported up to 35% [5]. Early literature reported that 50% of the patients who died had coexisting bacterial infection [6]. This is higher than what was previously seen during the influenza pandemic in 2009 when 25% of patients with influenza infection had secondary bacterial co-infection [7].

SARS-CoV-2 is a single stranded RNA Betacoronavirus and belongs to a corona virus family called Coronaviridae [8]. Phylogenetic analysis has revealed that SARS-CoV-2 is closely related to SARS-CoV-1 and genetically distinct from Middle East Respiratory Syndrome Coronavirus (MERS-CoV) [9]. SARS-CoV-2 utilizes Angiotensin-Converting Enzyme 2 (ACE-2) receptors in the lower airways which are also cellular receptors for other viruses in this group i.e. SARS-CoV and MERS-CoV [10]. Despite similar expression of ACE-2 receptors in different organs of the body, the most affected site is the lung tissue [11]. Influenza strains also cause lung damage by ACE-2 receptor mediated effects [12]. On the other hand, since the ACE-2 receptor used by SARS-CoV-2 is an interferon‐stimulated gene, it was hypothesized that type I and III interferons produced after bacterial infection may facilitate SARS‐CoV‐2 attachment [13].

Influenza A virus (IAV), a member of the Orthomyxoviridae, is a respiratory pathogen. The genome consists of eight segmented negative-sense RNAs. The viral ribonucleoproteins (vRNPs) consist of replicase complex (PA, PB1, and PB2) and various viral nucleoproteins (NPs) to form viral ribonucleoproteins (vRNPs). Newly assembled vRNPs may also contain matrix 1 (M1) and nuclear export protein (NEP). The lipid bilayers of influenza A virus particles contain three viral integral membrane proteins, hemagglutinin (HA), neuraminidase (NA), and matrix 2 (M2) [14]. The HA, NA, M1, and M2 proteins are important for influenza A virus assembly and budding [15]. Amino acid changes in the M1 protein of influenza viruses are known to increase virus budding to add lethal mutations in the M2 component in the cytoplasmic tail [16].

During pandemics, the detection of the novel virus may lead to underreporting of other pathogens that could be the etiological agent contributing to the disease severity. Indeed, during the influenza A (H1N1) pdm09 pandemic, 44.3% of patients had unreported respiratory viruses [17]. Earlier studies indicated that common viral co-infections reported in COVID-19 patients include Influenza viruses, RSV and adenovirus [5, 18]. Bacterial co-infections is more frequent than viral co-infections and it is homogeneously distributed in mild, moderate or severe illness [19]. The commonly known COVID-19 co-infecting bacteria are *Mycoplasma pneumoniae*, *Pseudomonas aeruginosa*, *Heamophilus influenzae* and *Chlamydia pneumoniae* [20]. These findings clearly emphasize the importance of screening for other clinically important co-circulating respiratory pathogens contributing to the etiology of the disease.

The novelty of SARS-CoV-2 and the complexity of profound etiology of co-infection urged for consideration of comorbidities. COVID-19 patients with an underlying condition such as hypertension, diabetes, chronic kidney disease, and heart failure have been associated with COVID-19 disease severity [21]. Cardiovascular disease has a strong association with COVID-19 pneumonia (14.4%) [7, 21] and other common comorbidities found in patients with SARS-CoV-2 include hypertension (18.6%) and diabetes (11.9%) [22]. Comorbidities were also linked with increased hospitalization, prolonged stay in ICU, and mortality. Hypertension was more prevalent in severe cases (47%) compared to diabetes (24%) and Respiratory diseases (10%) among other underlying conditions [21].

In conclusion, extensive evidence revealed that viral infections predispose patients to subsequent bacterial co‐infections [7]. This knowledge gap is puzzling as limited number of reports have described prevalence of bacterial and viral co-infections simultaneously. We hypothesized that undetected co-infections might have severe clinical implications associated with increased hospitalization, prolonged stay in ICU, and mortality. Therefore, our aim was, to investigate the presence of viral and bacterial co-infections in ICU and non-ICU COVID-19 patients.

## Methods

### Patients' demography

The study specimens were obtained retrospectively from a population (n = 48) admitted to King Fahad Hospital, Medina, Saudi Arabia. The number of critical cases needing admission to the ICU was 14, and 34 were mild cases. Nine patients died, (all were admitted to the ICU), and the rest survived. Thirteen patients were Saudi citizens and the rest were non Saudis (Table 1).

**Table 1:** Demographics and clinical characteristics of COVID-19 patients

Baseline variables	All patients	Non-ICU	ICU	P-value
	(N = 48)	N = 34 (71%)	N = 14 (29%)	
*Demographics*				
Age				
Median	52 ± 18	46 ± 18	62 ± 15	
Range	1–92	1–92	25–74	
Gender				
Men	37 (77%)	26 (70%)	11 (30%)	p = 0.87*
Women	11 (23%)	8 (73%)	3 (27%)	
*Characteristics*				
Co-infection				
Co-infection	34 (71%)	25 (74%)	9 (26%)	p = 0.52*
No co-infection	14 (29%)	9 (64%)	5 (36%)	
Multiple co-infections	11 (32%)	9 (82%)	2 (18%)	p = 0.49*
Single co-infection	23 (68%)	16 (71%)	7 (29%)	
Case fatality rate	9 (19%)	–	9 (100%)	
*Comorbidities*				
Cardiovascular disease	2 (4%)	0 (0%)	2 (100%)	p = 0.22*
Chronic kidney disease	5 (10%)	4 (80%)	1 (20%)	p = 0.63*
Diabetes	26 (54%)	22 (85%)	4 (15%)	p = 0.02**
Others	13 (27%)	6 (46%)	7 (54%)	p = 0.02**
Serological markers (median)				
d-dimer (0–0.5)	1.6	1.7	1.4	p = 0.25*
LDH (98–192)	336	335	349	p = 0.12*
Troponin T (0–0.07)	0.01	0.01	0.05	p < 0.05**

*Not significant at p < 0.05; **Significant at p > 0.05.

### RNA extraction and SARS-CoV-2 PCR detection

The handling of respiratory samples, preparation of aliquots and viral RNA extraction were performed in accordance with relevant guidelines and regulations. SARS-CoV-2 RNA was extracted by MagNA Pure 96 instrument, using the MagNA Pure 96 DNA and Viral NA small volume kit, (Roche, Germany). Reverse Transcription Polymerase Chain Reaction (RT-PCR) was performed by Roche LightCycler® 480 II instrument, using the RNA Process Control Kit Trial Pack (Roche, Germany) with an internal, positive, and negative controls.

### Real time PCR confirmation of SARS-CoV-2

RT-PCR assay targeting the E gene and RdRp (ORF1ab) of SARS-CoV-2 was carried out in 7500 Fast Real-Time PCR instrument (Thermo Fisher scientific, USA) with the Superscript III one-step RT-PCR kit (Invitrogen, Darmstadt, Germany) and performed as previously described [23, 24]. Thermal cycling conditions were 55 °C for 10 min, then 95 °C for 3 min, followed by 45 cycles of 95 °C for 15 s, and lastly 58 °C for 30 s.

The primers used for RdRp gene were as follows: RdRp_SARSr-F (GTGARATGGTCATGTGTGGCGG), RdRP_SARSr-P1: (FAM-CCAGGTGGWACRTCATCMGGTGATGC-BBQ) and RdRp_SARSr-R: (CARATGTTAAASACACTATTAGCATA). The primers used for E gene were as follows: E_Sarbeco_F (ACAGGTACGTTAATAGTTAATAGCGT), E_Sarbeco_P1 (FAM-ACACTAGCCATCCTTACTGCGCTTCG-BBQ) and E_Sarbeco_R: (ATATTGCAGCAGTACGCACACA).

### Real time PCR panel for co-infections

The quantitative Real-Time PCR assay for the co-infecting respiratory pathogens was performed on 7500 Fast Real-Time PCR instrument (Thermo Fisher scientific, USA). Extracted RNA was reinvestigated by RT/q-PCR with Fast Track Diagnostic (FTD) Respiratory pathogens 21 plus kit (Biomerieux, Luxemburg) according to the manufacturer’s instructions using 6 multiplex PCR amplifications for respiratory viruses and bacteria. The included viruses were Flu A (H1N1); Flu B; rhinovirus; seasonal Coronaviruses NL63, 229E, OC43 and HKU1; parainfluenza 1, 2, 3 and 4; metapneumoviruses A and B; bocavirus; RSV A and B; adenovirus; enterovirus and parechovirus. The screened bacteria were *Chlamydia pneumoniae*; *Mycoplasma pneumoniae*; *Staphylococcus aureus*; *Streptococcus pneumoniae* and *Heamophilus influenzae* B. This multiplex PCR assay also included six positive controls: five for each viral panel and one for bacterium. It also incorporated six negative controls provided in the kit. Briefly, 10 µl of the extracted RNA was added to 12.5 μl of buffer, 1 μl of enzyme, 2 pmol of each primer and probe (corresponding to each pathogen). Thermal cycling conditions include reverse transcription for 15 min at 42 °C, denaturation for 3 min at 94 °C followed by 40 cycles of primers' annealing for 8 s at 94 °C, and 34 s of template extension at 60 °C. Positivity or negativity of results were determined according to the manufacturer’s interpretation guide, and 12 samples were randomly chosen and repeated for re-confirmation purpose.

### Data collection

Demographic and clinical data (Table 1) were obtained without any personally identifiable information, including the following clinical laboratory results: age, gender, history of chronic illness, Ct value, d-dimer, CK, CK-MB, Trop, HB, Platelet, RBC, WBC, Neutrophil, Lymph, CRP, Pro calciponin, Glucose, ESR, LDL, AST, ALT, Urea, Creatinine, LDH, Albumin, Total Protein and blood group. The clinical datasets used and analyzed during this study are available from the corresponding author on request.

### Statistical analysis

Minitab version 19.0 software was used for statistical analysis. All data was expressed as continuous variables. Continuous data was expressed by median for normally distributed variable i.e. age; whereas absolute numbers were expressed as percentages. The paired t-test was used to compare continuous variables of normal distribution and non-normal distribution, respectively. We used Pearson Coefficient to show association between the variables. Relationship of one response and multiple predictors was examined using linear regression with best fitted model. Mortality was evaluated for bacterial and viral co-infections by using binary logistic expression and expressed as odds ratio. MANOVA (multivariate analysis of variance) was used to analyze mortality in the presence of co-infection and comorbidities and it was expressed as p-value. The patients were grouped by disease severity, comorbidities and co-infection or not. Factors were adjusted for age and gender. A 2-sided α of less than 0.05 was considered statistically significant.

## Results

**Fig. 1:**
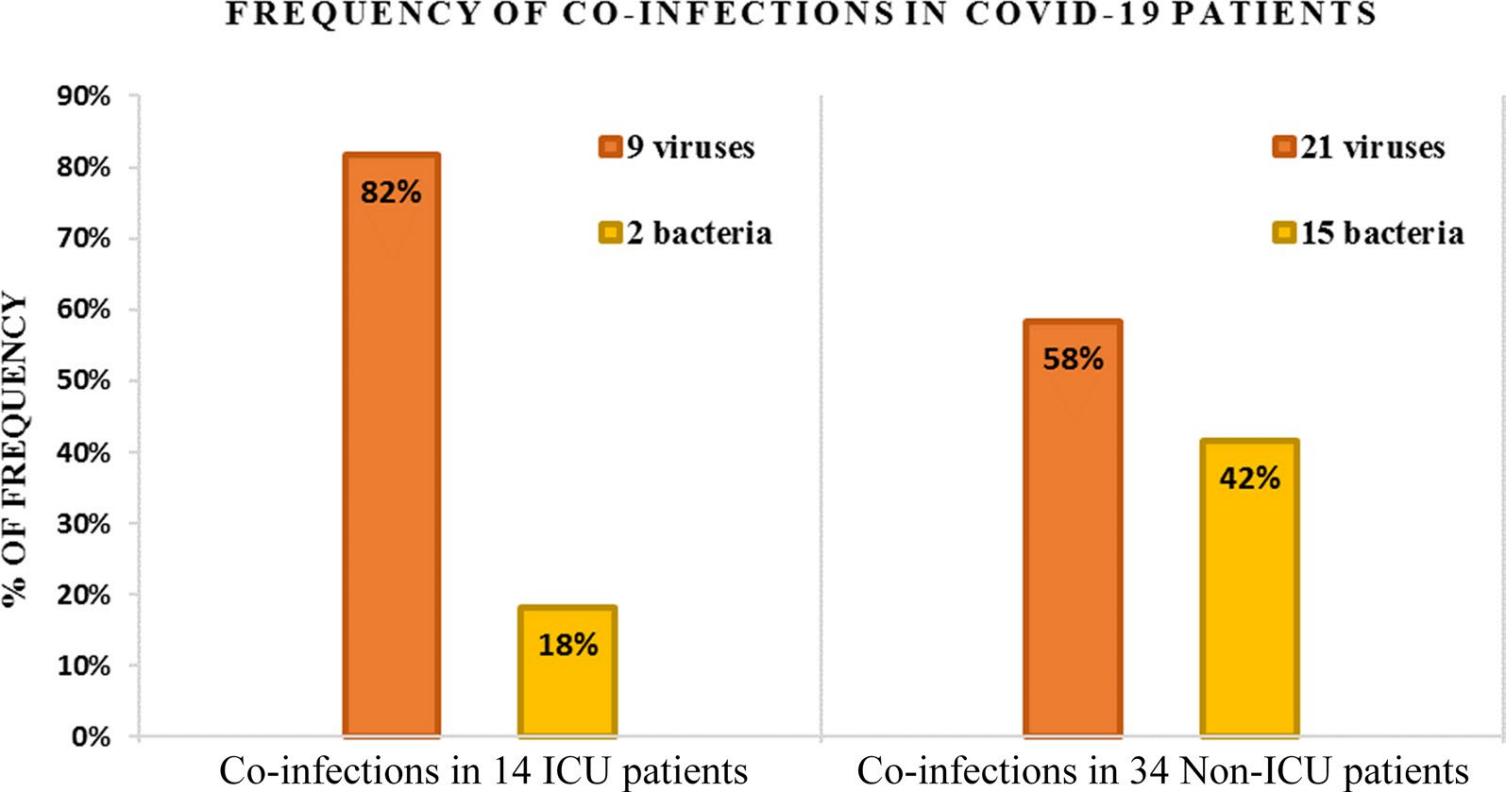
Frequency of coexistence of pathogens in COVID-19 patients. The figure shows the frequency of viral vs bacterial co-infections in COVID-19 patients. The number of viruses detected in 14 ICU patients was 9 (6 H1N1 and 3 Adenovirus) compared to 2 bacteria (1 *Chlamydia pneumoniae* and 1 *Staphylococcus aureus*) which indicates a higher likelihood of ICU admission with viral co-infection. In 34 non-ICU patients, 36 coexisting pathogens were detected namely 15 bacteria (12 *Chlamydia pneumoniae* and 3 *Staphylococcus aureus*) and 21 viruses (11 H1N1, 7 Adenovirus, 1 metapneumovirus, 1 parainfluenza-3, and 1 influenza B) although none of them were involved in mortality

We investigated co-infection in 48 COVID-19 patients (including 37 males and 11 females), the male to female ratio was 3:1. Median age of our study population was 52 years (1–92). Fourteen patients (29%) needed admission to intensive care unit (ICU cases). The remaining 34 patients (71%) did not require ICU admission and were classified as non-ICU cases. We found co-infections in thirty-four (71%) patients. Although severity of disease was negatively correlated (r = -0.09) with the presence of a co-infection (p = 0.53), it had a positive correlation with the co-infecting viruses (r = 0.1, p = 0.42) by Pearson Coefficient as shown in Fig. 1. Furthermore, statistically significant inverse association was observed (r = -0.28, p = 0.04) between bacterial co-infection and ICU admission. In other words, this association indicates a lower likelihood of ICU admission with bacterial co-infection (Fig. 1). The most commonly found co-infecting virus was influenza A H1N1 in 17 patients (36%). *Chlamydia pneumoniae* was the most prevalent co-infecting bacteria found in 13 patients (28%). Other organisms detected were adenovirus in 10 patients and S. aureus in 4 patients. It was noticed that 4/17 (23.5%) patients with H1N1 had coexisting *Chlamydia pneumoniae* suggesting that 62% (21/34) may have triple co-infection.

**Fig. 2:**
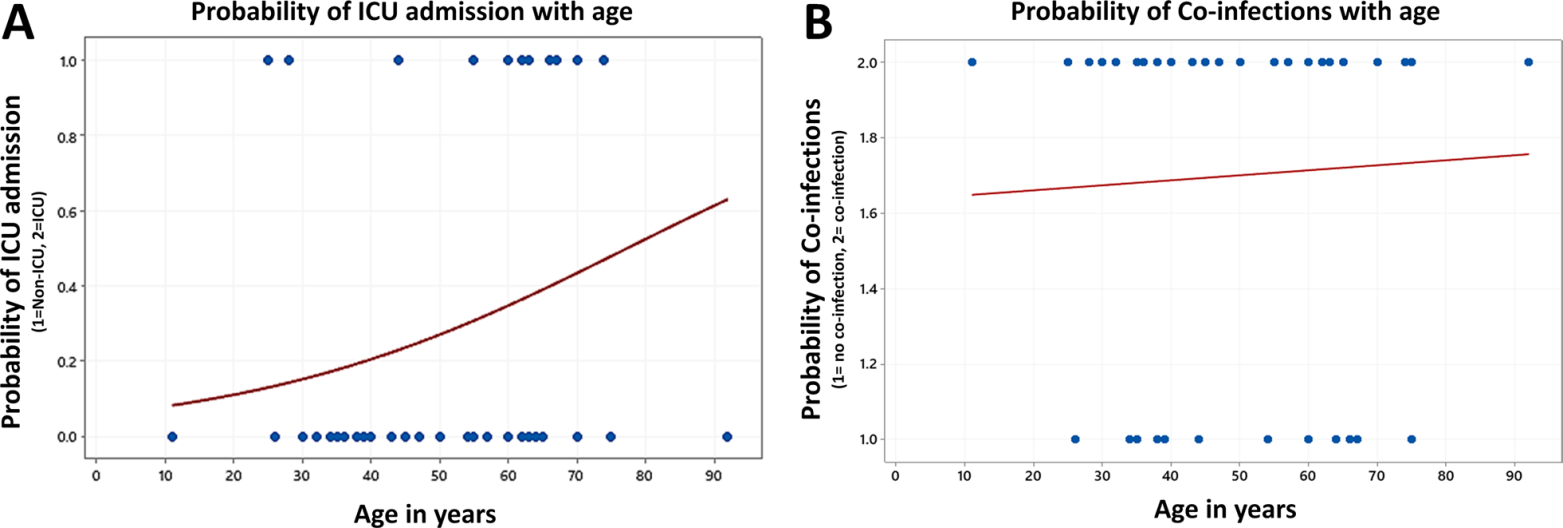
The binary fitted line plot shows the correlation between age and the probability of ICU admission and co-infection among COVID-19 patients. (**A**) The probability of an admission into the ICU among different age groups revealed an increasing trend with aging. (**B**) The probability of detection of a coinfection also exhibited a moderate linear correlation with age

Binary logistic regression was used to analyze the mortality association with viral and bacterial co-infections. The mortality rate was 19% (9/48), all of them were critically ill COVID-19 patients admitted to the ICU, and two thirds of SARS-CoV-2 critically ill patients who died had a co-infection (6/9). The likelihood of an admission into the ICU and the probability of detecting a co-infection among different age groups revealed an increasing trend with aging (Fig. 2). We found that viral co-infections (OR = 1.78, CI = 0.38–8.28) had higher mortality compared to bacterial co-infections (OR = 0.44, CI = 0.08–2.45) in COVID-19 patients. We also observed that there was a positive correlation between co-infecting influenza H1N1 virus and mortality (r = 0.2). On the other hand, co-infection with *Chlamydia pneumoniae* (r = -0.17) did not have any correlation with mortality in SARS-CoV-2 infected patients (Table 2).

**Table 2:**
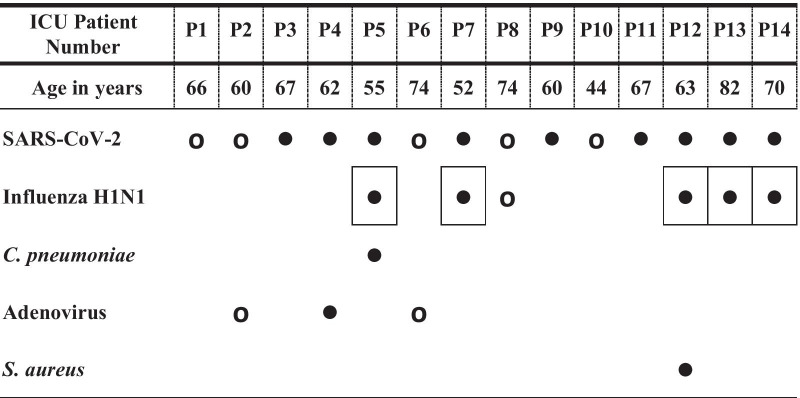
Distribution of SARS-CoV-2 and other respiratory co-infections among 14 ICU patients (P). The table shows the age and distribution of 11 co-infecting pathogens in 9 out of a total of 14 ICU patients. In addition to SARS-CoV-2, 8 coexisting pathogens were detected in 6 deceased patients (●), while the remaining 3 fatalities (P3, P9, and P11) tested negative for all investigated pathogens. Remarkably, influenza A H1N1 was associated with the death of 5 of those patients (squared)

Closed circle (●) represents deceased patients. Open circle (ο) represents survived patients

In terms of comorbidities, the prevalence of diabetes was 54% (26/48), cardiovascular disease 4% (2/48) and chronic kidney disease (CKD) 10.4% (5/48). Other comorbidities including lobar pneumonia, cancer, acute kidney failure, and rheumatoid arthritis were found in 13 (27%) of patients despite the occurrence of multiple comorbidities in one patient. There was no significant association of co-infection with diabetes (p = 0.25), cardiovascular disease (p = 0.24) nor CKD (p = 0.7). However, when we used MANOVA test to look at associations of death and co-infection with diabetes, cardiovascular disease or CKD, it showed (Table 1) that statistically significant correlation was present only between diabetes and death (p = 0.02).

We also investigated the importance of different blood markers in COVID-19 patients (Table 1). Specifically, we examined association of d-dimer, lactate dehydrogenase (LDH) and Troponin T with the severity of disease. These markers have been interchangeably used to predict disease severity and the potential of ICU admission. Using linear regression, we found that Troponin T was strongly related (p = 0.001) with disease severity compared to LDH (p = 0.12) and d-dimer (p = 0.25). This finding may imply that Troponin T could be used as a predictor for disease severity.

## Discussion

In this study, we investigated the presence of co-infections in COVID-19 cases and analyzed their clinical and epidemiological characteristics. We found evidence of co-infection in 34 COVID-19 patients (71%). Influenza A H1N1 was the most common detected among the co-infecting viruses found in 64% of patients. Viral and/or bacterial co-infections have been linked to disease severity, both directly, indirectly and through immunological response [25, 26]. Several studies have partially reported the prevalence of COVID-19 pneumonia and influenza co-infection [27–29]. However, data on clinical significance of influenza A H1N1 co-infections with COVID-19 is limited. The similarity of clinical manifestations between the circulating respiratory viruses such as influenza A H1N1 and SARS-CoV-2 makes the differentiation very difficult [30, 31]. Influenza A H1N1 dominance in our study population implies simultaneous spread of two viruses and clearly emphasizes on the importance of screening for other clinically important co-circulating respiratory pathogens. Besides, numerous studies have shown viral co-infections being associated with disease severity, acute respiratory distress syndrome (ARDS) and even death. These studies show higher intensive care admission rates in patients presenting with co-infections [5, 28, 32]. In this study, influenza A H1N1‐COVID‐19 co-infected cases were more severe and required ICU admission. Our results also showed a high case fatality rate among COVID-19 viral co-infections (r = 0.2) as two thirds of SARS-CoV-2 critically ill patients who died had co-infection (6/9). The severity and higher case fatality among COVID‐19 viral co-infected patients may be attributed to influenza A H1N1, which is known to induce a strong inflammatory cytokine/chemokine response (cytokine storm). Thus, the H1N1‐COVID‐19 co-infection could accelerate and play a major role in ARDS development. Our data showed that, during pandemics, focusing on the detection of the novel virus may lead to underreporting of other pathogens that could be the etiological agents contributing to disease severity.

The reporting of COVID‐19 and Influenza co-infection in the literature has been low despite high rates of single infection. Some studies have reported the difficulty in differentiating clinical characteristics between the co-infection of SARS‐CoV‐2 and Influenza [33, 34]. Regular SARS‐CoV‐2 respiratory screening is not sufficient to rule out the possibility of co-infection. The influence of the ongoing COVID‐19 pandemic should enhance utilization of a better diagnostic approach and testing of other respiratory pathogens.

Unfortunately, the topic of co-infection is usually embedded within the characteristics of patients in COVID-19 studies. However, our study investigated the coexistence of a full panel of respiratory viruses and bacteria simultaneously in order to investigate whether viral infection predisposes patients to subsequent bacterial co‐infection or not. Indeed, secondary bacterial co-infection is identified as the main cause of death in patients with viral pneumonia [7, 35]. A study of common respiratory pathogens presenting as co-infections with COVID-19 from China revealed that the *Mycoplasma pneumoniae* and *Legionella pneumophila* were the most common bacteria detected among COVID-19 patients [36]. In this study, bacterial co-infection was present in 36% of patients and the most common bacterial co-infection among COVID-19 patients was *Chlamydia pneumoniae* with infection rate of (27%). Our findings appeared to be inconsistent with previous findings from China [36] which could be attributed to the diversity of geographical distribution of circulating respiratory bacteria. Nevertheless, the *Chlamydia pneumoniae* infection is a common cause of acute respiratory infections with seroprevalence of 34.1% in patients with fatal COVID-19 [37]. However, inverse association was observed between bacterial co-infection and disease severity. This association indicates a lower likelihood of ICU admission with bacterial co-infection which may be attributed to empirical use of antibiotics during the early onset of COVID-19 disease. It could be argued that COVID-19 patients co-infected with *Chlamydia pneumoniae* who are treated with antibiotics may have suppressed the opportunistic growth of potentially fatal secondary bacterial infections decreasing the likelihood of ICU admission.

Many risk factors including older age, diabetes mellitus, cardiovascular disease, elevated LDH levels, high levels of d‐dimer and elevated inflammatory cytokines/ chemokines have been associated with adverse outcomes in COVID-19. In our study, the prevalence of diabetes was 54% and significant correlated was present between death and co-infection with diabetes (p = 0.02). This is expected as poor glycemic control predisposes to impaired innate and adaptive immunity which might lead to decreased viral clearance [38]. The Troponin complex is a predictor for coronary syndrome and myocardial infarction. The high levels of Troponin are significantly associated with acute myocardial infarction [39]. In this study, high levels of Troponin T were detected among COVID-19 patients. We found that Troponin T was strongly related (p = 0.001) with disease severity compared to LDH (p = 0.12) and d-dimer (p = 0.25). This is explained by presence of ACE-2 receptors on myocardial cells and presence of myocardial injury in SARS-CoV-2 infection [40]. Several studies have revealed that the higher Troponin levels were increased in COVID-19 patients' ICU admission and in-hospital death [19, 41, 42]. Our results confirm the important role of Troponin in the COVID-19 severity. We think the Troponin levels can be used as a marker of COVID-19 severity and a predictor of cardiovascular events.

There have been few reports since the start of pandemic about interaction of influenza and COVID-19 most probably due to social distancing causing low incidence of influenza viruses [31]. Other studies have indicated the severity of infection in patients found to be co-infected with influenza and SARS-CoV-2 [43] and surprisingly mild symptoms in outpatients having co-infection [44]. Similar to our study, Wuhan, China in particular has seen a tremendous increase (57.3%) in the incidence of influenza and SARS-CoV-2 co-infection [45]. Therefore, both SARS-CoV-2 and influenza virus co-infection coupled with variable clinical prognosis poses a greater challenge from public health stand point. Our study showed a high case fatality rate among COVID-19 viral co-infections (r = 0.2). The severity and higher case fatality among COVID‐19 viral co-infected patients could be attributed to influenza A co-infection, since it was recently reported that SARS-CoV-2 infection followed by influenza both in-vivo and in-vitro increased the severity of infection [46].

Our study has some limitations. First, only 48 COVID-19 patients were included. Second, our study did not include asymptomatic or pre-symptomatic cases or healthy non- COVID‐19 controls. Future studies to overcome these limitations need to be considered.

## Conclusions

In conclusion, the similarity in clinical presentation for both COVID-19 and Influenza makes it difficult to assess their impact on ICU admission and mortality. Our study highlights the importance of screening for co-infecting viruses in COVID-19 patients, given the high prevalence of Influenza viruses. The detection of co-infections in COVID-19 cases shows the importance of flu vaccination and warrants its increased coverage to reduce the hospitalization and associated mortality.

## Data Availability

The datasets used and/or analyzed during the current study are available from the corresponding author on reasonable request.

## Abbreviations

COVID-19: Coronavirus Disease-19
ICU: Intensive Care Unit
OR: Odd Ratio
SARS-CoV-2: Severe Acute Respiratory Syndrome Coronavirus 2
MERS-CoV: Middle East Respiratory Syndrome Coronavirus
ACE-2: Angiotensin-Converting Enzyme 2
Pdm09: The 2009 H1N1 Pandemic
LDH: Lactate Dehydrogenase
RT-PCR: Reverse Transcription Polymerase Chain Reaction
CK: Creatine Kinase
Trop: Troponin
HB: Hemoglobin
RBC: Red Blood Cell
WBC: White Blood Cells
CRP: C-Reactive Protein
ESR: Erythrocyte Sedimentation Rate
LDL: Low-Density Lipoproteins
AST: Aspartate Aminotransferase
ALT: Alanine Aminotransferase
CKD: Chronic Kidney Disease
ARDS: Acute Respiratory Distress Syndrome
IRB: Institutional Review Board
